# Eugenol improves salt tolerance via enhancing antioxidant capacity and regulating ionic balance in tobacco seedlings

**DOI:** 10.3389/fpls.2023.1284480

**Published:** 2024-01-16

**Authors:** Jiaxin Xu, Tingting Wang, Changwei Sun, Peng Liu, Jian Chen, Xin Hou, Tao Yu, Yun Gao, Zhiguo Liu, Long Yang, Li Zhang

**Affiliations:** ^1^ College of Plant Protection, Shandong Agricultural University, Taian, China; ^2^ College of Horticulture, Nanjing Agricultural University, Nanjing, China; ^3^ Institute of Food Quality and Safety, Jiangsu Academy of Agricultural Sciences, Nanjing, China

**Keywords:** eugenol, oxidative stress, reactive oxygens species, redox homeostasis, salt stress, tobacco

## Abstract

Salt stress inhibits plant growth by disturbing plant intrinsic physiology. The application of exogenous plant growth regulators to improve the plant tolerance against salt stress has become one of the promising approaches to promote plant growth in saline environment. Eugenol (4-allyl-2- methoxyphenol) is the main ingredient in clove oil and it is known for its strong antioxidant and anti-microbial activities. Eugenol also has the ability of inhibiting several plant pathogens, implying the potential use of eugenol as an environmental friendly agrichemical. However, little is known about the possible role of eugenol in the regulation of plant tolerance against abiotic stress. Therefore, here we investigated the effectiveness of phytochemical eugenol in promoting salt tolerance in tobacco seedlings through physiological, histochemical, and biochemical method. The seedling roots were exposed to NaCl solution in the presence or absence of eugenol. Salt stress inhibited seedling growth, but eugenol supplementation effectively attenuated its effects in a dose-dependent manner, with an optimal effect at 20 µM. ROS (reactive oxygen species) accumulation was found in seedlings upon salt stress which was further resulted in the amelioration of lipid peroxidation, loss of membrane integrity, and cell death in salt-treated seedlings. Addition of eugenol highly suppressed ROS accumulation and reduced lipid peroxidation generation. Both enzymatic and non-enzymatic antioxidative systems were activated by eugenol treatment. AsA/DHA and GSH/GSSG were also enhanced upon eugenol treatment, which helped maintain redox homeostasis upon salinity. Eugenol treatment resulted in an increase in the content of osmoprotectants (e.g. proline, soluble sugar and starch) in salt-treated seedlings. Na^+^ levels decreased significantly in seedlings upon eugenol exposure. This may result from the upregulation of the expression of two ionic transporter genes, *SOS1* (salt-hypersensitive 1) and *NHX1* (Na^+^/H^+^ anti-transporter 1). Hierarchical cluster combined correlation analysis uncovered that eugenol induced salt tolerance was mediated by redox homeostasis and maintaining ionic balance in tobacco seedlings. This work reveals that eugenol plays a crucial role in regulating plant resistant physiology. This may extend its biological function as a novel biostimulant and opens up new possibilities for improving crop productivity in the saline agricultural environment.

## 1 Introduction

Soil salinity is one of the important environmental problems worldwide (Al-Turki et al., 2023; Xiao and Zhou, 2023). About 20% of the world’s irrigated land is affected by salinity (Wang et al., 2023b). Poor irrigation and industrial pollution continue to exacerbate soil salinization (Yu et al., 2020; Ge et al., 2023). It has been estimated that about 50% of the world’s arable land would be salinized in 2050 (Shrivastava and Kumar, 2015). Salt stress inhibits crop growth, leading to the decrease in crop yield (Guijarro-Real et al., 2020). Salt stress-induced phytotoxicity involves various plant physiological disorders. High salinity results in plant dehydration by inducing osmotic stress. Plants activate osmotic adjustment to combat salt stress (van Zelm et al., 2020). Plant cells tend to accumulate carbohydrates (e.g. sugar and starch) to maintain cell turgor under saline conditions (Wang et al., 2021). Proline is another important osmotic regulator (Patani et al., 2023). Proline accumulation plays vital roles in adjusting osmotic potential in plant cells upon salt stress (Mansour and Ali, 2017).

ROS (reactive oxygen species) overaccumulation is one of the typical consequences of salinity-induced phytotoxicity (Wang et al., 2023a). Basically, ROS comprises non-radical form (e.g. hydrogen peroxide, H_2_O_2_) and free radical form (e.g. superoxide radical, O_2_^·−^) (Gill and Tuteja, 2010). Salinity-induced ROS frequently attacks macromolecules in plant cells, resulting in oxidative stress and cell death (Liu et al., 2023). Accordingly, plants detoxify excessive ROS by deploying endogenous antioxidative systems, such as enzymatic system including a set of antioxidative enzymes (e.g. superoxide dismutase, SOD; peroxidase, POD; catalase, CAT; and ascorbate peroxidase, APX) and non-enzymatic system including various antioxidants (e.g. AsA, ascorbic acid; GSH, glutathione; and proline) (Hasanuzzaman et al., 2021). GSSG and DHA (dehydroascorbic Acid) are the oxidized form of GSH and AsA, respectively (Luo et al., 2023). The redox balance can be modulated by GSH/GSSG and AsA/DHA, which play a role in plant responses to abiotic stress (Kumari et al., 2023). Promoting plant salt tolerance has been closely associated with the enhancement of antioxidant capacity and the maintenance of redox status (Gao et al., 2022).

Plant salt tolerance is also involved in maintaining intracellular ionic homeostasis (Song et al., 2021; Zhang et al., 2023b). In saline conditions, excessive solidum (Na^+^) in cytosol are toxic to plant cells (Xiao and Zhou, 2023). Plants activate SOS (Salt Overly Sensitive) pathway to prevent Na^+^ accumulation in cytosol. In this pathway, *SOS1* is a Na^+^ transporter localized in plasma membrane (Ma et al., 2022). *SOS1* excludes Na^+^ out of cells to detoxify Na^+^. *SOS2-SOS3* kinase complex can phosphorylate and activate *SOS1* (Ali et al., 2023). Plant cells also have another strategy to lower Na^+^ concentration in cytosol through Na^+^ sequestration in vacuole. For example, *NHX1* (*Na^+^/H^+^ antiporter 1*) encodes a transporter localized in tonoplast. It can compartmentalize Na^+^ into vacuole in order to alleviate Na^+^ toxicity in cytosol (Zhang et al., 2017). In Arabidopsis, *NHX1* also has the ability to transport K^+^ for plant K^+^ uptake to regulate cell turgor upon salt stress (Barragán et al., 2012). Maintaining the proper balance between Na^+^ and K^+^ is vital for plant tolerance against salinity (Negrão et al., 2017).

Applying exogenous regulators to promote plant salt tolerance has been considering as a promising approach to improve the performance of crops in saline environment (Quamruzzaman et al., 2021). Eugenol (C_10_H_12_O_2_, 4-allyl-2-methoxyphenol) is the main constituent of essential oil obtained from clove (Jia et al., 2020). Eugenol can also be produced by other plants (e.g. strawberry sweet basil and petunia), contributing to aroma (Koeduka et al., 2006; Aragüez et al., 2013). The clinical relevance of eugenol has been associated with its anti-inflammatory activity and antimicrobial activity (Taleuzzaman et al., 2021). As a natural bioactive compound, eugenol can be used as food preservative in food antisepsis field (Hu et al., 2018b). Eugenol inhibits the growth of several agricultural pathogens (Morcia et al., 2011; Olea et al., 2019), implying the potential application of eugenol in agriculture. There are limited reports for eugenol-regulated plant physiology. Several recent studies suggest that eugenol has the ability to modulate plant resistant physiology against abiotic stresses. Eugenol confers drought tolerance in tea plant and heavy metal tolerance in *Brassica rapa* by modulating antioxidant defense through hydrogen sulfide- and abscisic acid-mediated signaling, respectively (e.g. heavy metal, drought, and cold) (Hu et al., 2018a; Zhao et al., 2022). These reports imply the potentiality of using eugenol as an environmental-friendly agrichemical to induce plant tolerance. Till now, little is known about the possible role of eugenol in regulating plant physiology against salinity stress. Mining the capability and mechanism of eugenol-conferred plant salt tolerance would help extend the biological function of eugenol in agriculture.

Tobacco is not only an importantly economic crop but also one of the model plants to study plant physiological adaptation under environmental stimuli (including salt stress). Tobacco plant activates salt tolerant responses by deploying antioxidative system, ion transport system (Na^+^ and K^+^ transporters), and osmotic regulation. In addition, some transcription factors (TFs) are involved in salt tolerant responses in tobacco. These TFs consist of AP2/ERF, WRKY, and zinc finger proteins, working on the regulation of the expression of genes that help decrease ROS and adjust osmotic response. CDPKs (calcium-dependent protein kinase) and MAPK (mitogen-activated protein kinases) are also play a role in modulating salinity stress by regulating ROS and hormonal signaling in tobacco plants (Sun et al., 2020). In this work, we detected the ability of eugenol in alleviating salt stress in tobacco seedlings. Then we studied the possible role of eugenol in antioxidation, osmotic adjudgment, and ionic balancing in tobacco seedlings upon salt stress. Finally, we discussed the possible mechanism of eugenol driving these physiological processes and their significance.

## 2 Materials and methods

### 2.1 Plant culture and treatment

The seeds of tobacco (K326) were provided by the Germplasm Resources Laboratory of Shandong Agricultural University. Tobacco seeds were rinsed with distilled water followed by sterilized with potassium permanganate (0.2%) for 10 min. Then the seeds were washed with distilled water again for three times. The seeds were placed on moistened triple-layer filter paper in Petri dishes for germination and growth. All the seedlings were cultured in a plant growth cabinet with light intensity of 5000 lx, photoperiod of 12 h, relative air humidity of 50%, and temperature at 26°C. Sixty identical seedlings (4-days old) with root length of 0.5 cm were selected and transferred into a new Petri dish with different concentrations of NaCl (sodium chloride) (0, 50, 100, 150, 200, and 250 mM). Eugenol (0, 5, 10, 20, 40, and 80 µM) was added to the treatment solution according to different treatment groups. The seedlings were harvested for physiological analysis after treatment for 3 days. For the time-course experiment, the seedling samples were analyzed at 0, 12, 24, 36, 48, 60, and 72 h, respectively.

### 2.2 Measurement of root elongation and fresh weight

We measured the root length of seedlings before and after treatment, respectively. The root elongation was calculated as the difference between these two values. The average root elongation was obtained based on 10-20 replicates for each treatment. The seedlings after treatment were surface-dried gently with filter paper before weighing. Ten seedlings were weighed together as one replicate. The average fresh weight (per 10 seedlings) was obtained based on three replicates for each treatment.

### 2.3 ROS measurement

Seedlings after treatment were harvested and washed with distilled water. Then the seedlings were used for determining the content of ROS quantified by measuring H_2_O_2_ and O_2_^·−^. The seedling samples were ground and homogenized with cold sodium phosphate buffer (50 mM, pH 7.4), followed by centrifuging at 12, 000 g at 4°C for 20 min. Then the supernatant was collected for the quantification of H_2_O_2_ and O_2_^·−^. A commercial Kit (BC3595, Beijing Solarbio Science & Technology Co., Ltd., Beijing, China) was used to determine H_2_O_2_ content based on spectrophotometric measurement of the product from the reaction between H_2_O_2_ and titanium sulfate (OD_415 nm_). Another commercial kit (BC1290, Solarbio) was used to determine O_2_^·−^ content based on spectrophotometric measurement of reaction between O_2_^·−^ and hydroxylamine hydrochloride (producing NO_2_^−^ to further react with sulfanilamide and N-ethylenediamine) (OD_530 nm_) (Sun et al., 2022).

Total ROS in root tips were detected histochemically by using specific fluorescent probe DCFH-DA (2′,7′-dichlorofluorescein diacetate) based on our previously published method (Ye et al., 2017). The roots after treatment were incubated in DCFH-DA solution (10 µM) at 25°C for 20 min in darkness. Then the roots were washed with distilled water for the observation of DCF fluorescence by using a fluorescence microscope (ECLIPSE, TE2000-S, Nikon, Melville, NY, USA).

The O_2_^·−^ in leaves were stained with NBT (nitro-blue tetrazolium) (Ye et al., 2016). The leaves were harvested and incubated in NBT solution (6 mM) for 3 h under light at 25°C. NBT can react with O_2_^·−^ in leaves to produce formazan compound that is navy blue. Then the leaves were washed with distilled water followed by transferring to boiling ethanol for 20 min to remove the chlorophyll background. A stereoscopic microscope (SteREO Discovery.V8, ZEISS) was applied to observe and to photograph the leaves.

### 2.4 Thiobarbituric acid reactive substances measurement

A TBARS Content Assay Kit (BC0025, Beijing Solarbio Science & Technology Co., Ltd., Beijing, China) was used to determine TBARS content in seedlings based on spectrophotometric assay of the reaction between TBA (1,3-diethyl-2-thiobarbituric acid) and TBARS in the presence of TCA (trichloroacetic acid) (Sun et al., 2022). The TBARS in root tips was also evaluated histochemically by using specific fluorescent probe C11-BODIPY(581/591) (a lipid peroxidation sensor) (Drummen et al., 2002). The roots were incubated in C11-BODIPY(581/591) solution (10 µM) at 25°C in darkness for 10 min, followed by washing with distilled water and photographing C11-BODIPY fluorescence under a fluorescence microscope (ECLIPSE, TE2000-S, Nikon, Melville, NY, USA).

### 2.5 Detection of membrane integrity and cell death

Loss of membrane integrity in root tips was detected by using Evans blue staining (Ye et al., 2017). The roots were incubated in 0.025% Evans blue solution at 25°C for 60 min, followed by photographing under stereoscopic microscope (SteREO Discovery.V8, ZEISS).

Cell death in root tips were detected with specific fluorescent probe PI (propidium iodide) (Kellermeier et al., 2013). The roots were incubated in PI solution (20 µM) at 25°C in darkness for 15 min, followed by visualization and photographing under fluorescent microscope (ECLIPSE, TE2000-S, Nikon, Melville, NY, USA).

Trypan blue staining was used to detect cell death in leaves (Ye et al., 2016). The leaves were incubated in Trypan blue solution (10 mg/mL) for 3 h under light at 25°C. Then the leaves were washed with distilled water followed by transferring to boiling ethanol for 20 min to remove the chlorophyll, allowing the appearance of blue. Then the leaves were observed and photographed by using a stereoscopic microscope (SteREO Discovery.V8, ZEISS).

### 2.6 Assay of the activity of antioxidative enzymes

About 0.1 g of fresh seedling samples were homogenized with 2 mL of cold phosphate buffer (50 mM, pH 7.0). Then the mixture was centrifuged at 12, 000 g for 15 min (4°C). The supernatant was collected for the determination of enzymatic activity. The SOD activity was determined spectrophotometrically (OD_560 nm_) using a commercial kit (BC0170, Solarbio) based on the quantification of the inhibition of photochemical reaction of NBT (nitro-blue tetrazolium) in a reaction system with methionine and riboflavin. The CAT activity was determined spectrophotometrically (OD_240 nm_) using a commercial kit (BC0205, Solarbio) based on the decomposition of H_2_O_2_. The POD activity was determined spectrophotometrically (OD_470 nm_) using a commercial kit (BC0090, Solarbio) based on the oxidation rate of guaiacol in the presence of H_2_O_2_. The APX activity was determined spectrophotometrically (OD_290 nm_) using a commercial kit (BC0220, Solarbio) based on the oxidation rate of AsA in the presence of H_2_O_2_ (Chen et al., 2020b).

### 2.7 Measurement of metabolites

A commercial kit (BC1230, Solarbio) was applied to determine AsA content based on the oxidation rate of AsA catalyzed by AAO (ascorbic acid oxidase) (spectrophotometric measurement at OD_265 nm_). A commercial kit (BC1240, Solarbio) was applied to determine DHA content based on the production rate of AsA from DHA by DTT (1,4-dithiothreitol) (spectrophotometric measurement at OD_265 nm_). A commercial kit (BC1170, Solarbio) was applied to determine GSH content based on spectrophotometric measurement of the product from the reaction between GSH and DTNB (5,5’-dithiobis-2-nitrobenoic acid) (OD_265 nm_). A commercial kit (BC1180, Solarbio) was applied to determine GSSG content based on the production rate of GSH (measurement of OD_265 nm_ for the reaction between GSH and DTNB) from GSSG catalyzed by GR (GSH reductase). A commercial kit (BC0290, Solarbio) was applied to determine proline content based on spectrophotometric measurement of the product from the reaction between proline and ninhydrin (OD_520 nm_). A commercial kit (BC0035, Solarbio) was applied to determine plant soluble sugar content based on anthrone colorimetric method (OD_620 nm_). A commercial kit (BC0700, Solarbio) was applied to determine starch content based on the separation of starch with 80% ethanol and acid hydrolyzation to glucose, followed by anthrone colorimetric method (OD_620 nm_) (Chen et al., 2020b).

### 2.8 Measurement of Na^+^ and K^+^ in seedlings

Dried seedling samples were digested with H_2_SO_4_ (with 10% H_2_O_2_) using microwave digestion system (MARS6, CEM Corporation, Matthews, NC, USA). Then the content of Na^+^ and K^+^ were determined by using atomic absorption spectrometer (PinAAcle 900T, PerkinElmer, Hopkinton, Massachusetts, USA).

### 2.9 Gene expression analysis

Total RNA was extracted from seedling samples using a plant RNA extraction kit (RNE05, Nobelab Biotech. Co., Ltd, Beijing, China). The first cDNA strand was synthesized from 1 μg of total RNA by using RescriptTM II RT SuperMix Reverse Transcriptase (Nobelab Biotech. Co., Ltd, Beijing, China). The qPCR (real-time quantitative polymerase chain reaction) was performed on a CFX96 Touch Real-Time PCR System (Bio-Rad, CA, USA) by using 2 × SYBR Premix UrTaq II (Nobelab Biotech. Co., Ltd, Beijin, Beijing, China). The relative abundance of *NtActin* was determined as internal standard to normalize the expression level of target genes. The primers used for amplifying the genes are as follows: *NtSOS1*, forward 5′-TGGAGGAAGCGACCGATTC-3′ and reverse 5′-CGATAACGAGAAGAGCGACAG-3′; *NtNHX1*, forward 5′-AAGAAGGTCATACTCAGTT-3′ and reverse 5′- GGTAGCAATAGTCTAATCAAT-3′; *NtActin*, forward 5′-GAAGAAGGTCCCAAGGGTTC-3′ and reverse 5′-TCTCCCTTTAACACCAACGG-3′.

### 2.10 Data analysis

Each result was shown as the mean ± standard deviation (SD) of 3 to 10 replicates. LSD (least significant difference) test was performed on the original data following ANOVA (one-way analysis of variance) tests to analyze the significant difference at p<0.05 among different treatments (SPSS 25.0, SPSS Inc., Chicago, IL, USA). The heatmap for hierarchical cluster analysis was generated by using TBtools (Chen et al., 2020a). Pearson correlation analysis was performed by using the package “corrplot” in R (Wei and Simko, 2017).

## 3 Results

### 3.1 Eugenol rescued the growth of tobacco seedlings under salt stress

**Figure 1:**
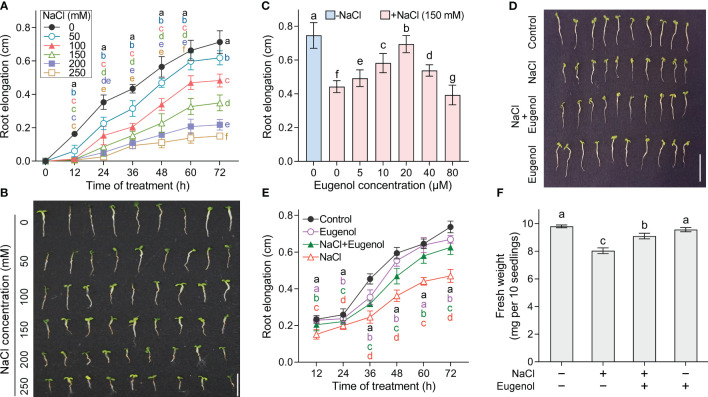
Effect of eugenol on the growth of tobacco seedlings upon NaCl exposure. **(A)** Time-course monitoring of root elongation under NaCl stress (0-250 mM). n = 9. **(B)** The seedling photograph taken after NaCl treatment for 72 hours. **(C)** Effect of eugenol (0-80 μM) on root elongation under NaCl (150 mM) stress for 72 h. n = 10. **(D)** Effect of eugenol at 20 μM on the seedling growth under NaCl (150 mM) stress for 72 h. **(E)** Time-course monitoring of root elongation under NaCl (150 mM) + eugenol (20 μM) treatment. n = 20. **(F)** Effect of eugenol at 20 μM on seedling fresh weight growth under NaCl (150 mM) stress for 72 h. n = 3. Different lowercase letters indicate significant difference among different treatments (p < 0.05, LSD).

Salt stress inhibited the root elongation of tobacco seedlings in time- and dose- dependent manner. NaCl at 50-250 mM began to inhibit root elongation at 12 h, followed with continuous decrease in the growth speed of roots (**Figure 1A**). Compared to the control group, the root elongation significantly decreased by 13.7%, 33.1%, 51.3%, 69.6%, and 79.1% upon NaCl exposure at 50, 100, 150, 200, and 250 mM, respectively, at the end of the exposure (72 h) (**Figures 1A, B**). We selected NaCl at 150 mM in the following experiments as it inhibited root elongation about half of control.

Compared to NaCl treatment alone, adding eugenol at 5-40 µM significantly rescued root elongation under NaCl treatment. Eugenol at 20 µM showed the greatest effect on root growth, resulting in significant increase in root elongation by 57.1% as compared to NaCl treatment alone (**Figures 1C, D**). Eugenol at 20 µM began to promote root elongation after treatment for 12 h, followed by enhanced growth speed of roots as compared to NaCl treatment alone (**Figure 1E**). Eugenol at 20 µM also significantly enhanced the fresh weight of seedlings under NaCl stress (**Figure 1F**). Eugenol treatment alone slightly affect the growth of tobacco seedlings (**Figures 1D–F**).

### 3.2 Eugenol alleviates salt-induced oxidative injury in tobacco seedlings

**Figure 2:**
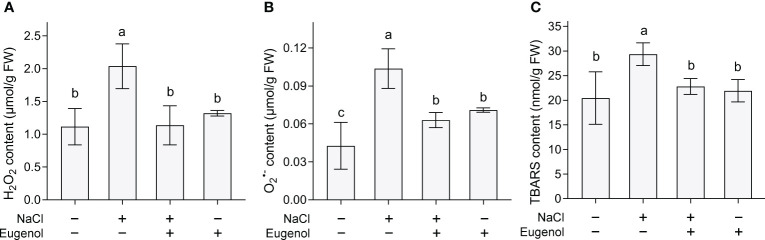
Effect of eugenol on the content of H_2_O_2_, O_2_^·−^, and TBARS contents in tobacco seedlings under salt stress. **(A)** H_2_O_2_ content. **(B)** O_2_^·−^ content. **(C)** TBARS content. Different lowercase letters indicate significant difference among different treatments (n = 3, p < 0.05, LSD).

Salinity always induces ROS accumulation in plants. NaCl exposure significantly increased the content of H_2_O_2_ and O_2_^·−^ increased by 43.7% and 101.9%, respectively, in tobacco seedlings. Adding eugenol resulted in significant decrease in the content of H_2_O_2_ and O_2_^·−^ by 22.3% and 41.1%, respectively, in NaCl-treated seedlings (**Figures 2A, B**). Then we measured TBARS content, a typical indicator of ROS-induced oxidative injury. Eugenol decreased TBARS content in seedlings by 22.3% as compared to NaCl treatment alone than induced TBARS accumulation (**Figure 2C**).

**Figure 3:**
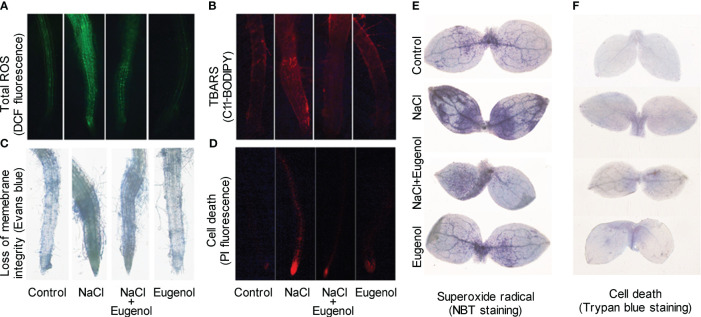
Histochemical detection of ROS and oxidative injury in tobacco seedlings upon NaCl and Eugenol treatment. **(A)** Total ROS in roots indicated by DCF fluorescence. **(B)** TBARS in roots indicated by C11BODYPI fluorescence. **(C)** Loss of membrane integrity in roots indicated by staining of Evans blue. **(D)** Cell death in roots indicated by PI fluorescence. **(E)** H_2_O_2_ content in leaves indicated by DAB staining. **(F)** Cell death in leaves indicated by Trypan blue staining.

We performed a set of *in vivo* detection to further verify eugenol-alleviated oxidative injury in tobacco seedlings. Salt stress induced extensive DCF fluorescence (indicating total ROS) and C11-BODIPY (indicating TBARS) in roots. Adding eugenol effectively decreased the fluorescence of these two probes, suggesting the decrease in the content of ROS and TBARS in roots (**Figures 3A, B**). ROS-induced lipid peroxidation results in membrane damage. The loss of membrane integrity was indicated by Evans blue staining. Adding eugenol decreased the staining of Evans blue in NaCl-treated root, suggesting that eugenol attenuated NaCl-induced membrane damage in root (**Figures 3C, D**). Superoxide radical (one typical ROS) in leaves was stained with NBT. Salt stress induced extensive NBT staining in leaf. Addition of eugenol showed slight NBT staining in the leaf of seedlings under salt stress (**Figure 3E**). Eugenol was able to attenuated salt-induced cell death in leaf indicated by Trypan blue staining (**Figure 3F**). All of these results suggested that eugenol alleviated NaCl-induced oxidative injury in tobacco seedlings.

### 3.3 Eugenol enhanced antioxidative capacity in tobacco seedlings under salt stress

**Figure 4:**
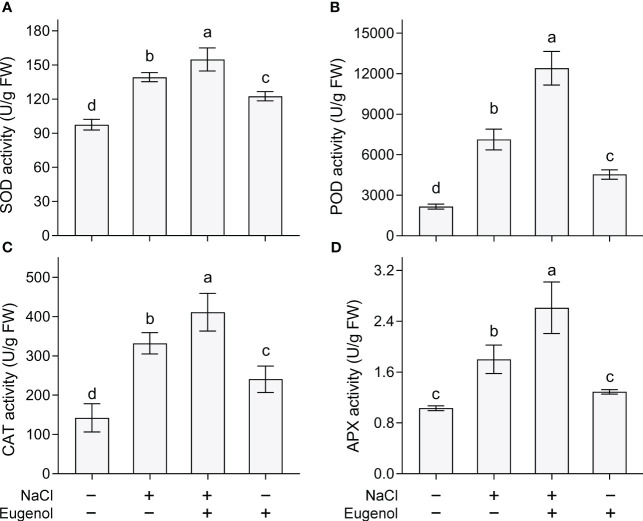
Effect of eugenol on the activities of antioxidant enzymes on tobacco seedlings under salt stress. **(A)** SOD activity. **(B)** POD activity. **(C)** CAT activity. **(D)** APX activity. Different lowercase letters indicate significant difference among different treatments (n = 3, p < 0.05, LSD).

Four typical antioxidative enzymes (SOD, POD, CAT and APX) were measured in tobacco seedlings. The activities of these enzymes were enhanced upon NaCl exposure. Compared to NaCl treatment alone, adding eugenol further significantly increased the activity of SOD, POD, CAT and APX by 11.2%, 74.0%, 44.9%, and 78.8%, respectively (**Figure 4**). Treatment with eugenol alone was able to increase the activities of SOD, POD, and CAT as compared to the control (**Figures 4A–C**).

**Figure 5:**
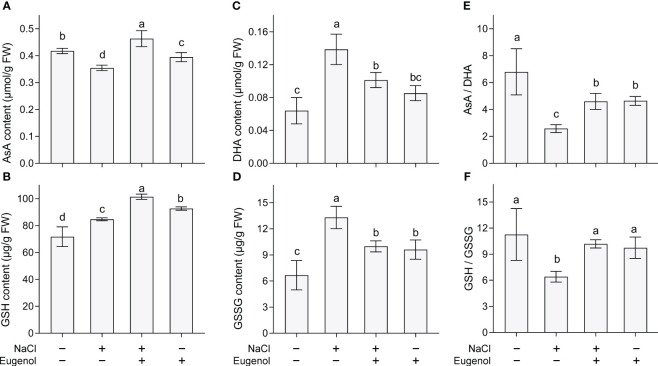
Effect of eugenol on antioxidants in tobacco seedlings under salt stress. **(A)** AsA content. **(B)** GSH content. **(C)** DHA content. **(D)** GSSG content. **(E)** The ratio of AsA to DHA. **(F)** The ratio of GSH to GSSG. Different lowercase letters indicate significant difference among different treatments (n = 3, p < 0.05, LSD).

AsA and GSH are two important antioxidants, belonging to non-enzymatic antioxidative system. Compared to NaCl treatment alone, adding eugenol significantly enhanced the content of AsA and GSH by 30.6% and 19.8%, respectively, in tobacco seedlings (**Figures 5A, B**). DHA and GSSG are the oxidized forms of AsA and GSH, respectively. Compared to NaCl treatment alone, adding eugenol significantly decreased the content of DHA and GSSG by 26.9% and 24.9%, respectively, in tobacco seedlings (**Figures 5C, D**). In addition, salt stress remarkably decreased the ratio of AsA/DHA and GSH/GSSG in tobacco seedlings, but adding eugenol significantly increased them by 78.3% and 58.9%, respectively (**Figures 5E, F**).

### 3.4 Eugenol enhanced the content osmotic metabolites in tobacco seedling under salt stress

**Figure 6:**
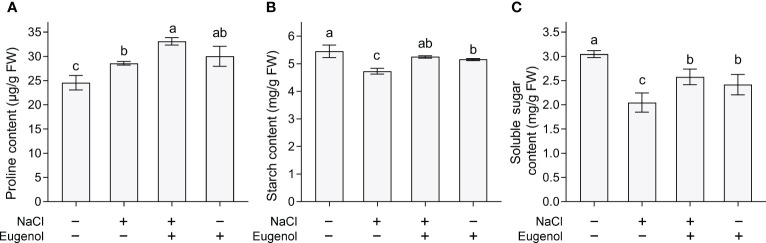
Effect of eugenol on the metabolites in tobacco seedlings under salt stress. **(A)** Proline content. **(B)** Starch content. **(C)** Soluble sugar content. Different lowercase letters indicate significant difference among different treatments (n = 3, p < 0.05, LSD).

In plant cells, proline is a metabolite regulating osmotic adaption induced by salt stress. Proline content increased proline in tobacco seedlings upon salt stress. Adding eugenol further enhanced proline content by 15.9% (**Figure 6A**). Compared to control, salt stress decreased the content of starch and soluble sugar in seedlings. Adding eugenol significantly the content of starch and soluble sugar by 26.9% and 24.9% (**Figures 6B, C**), respectively, as compared to NaCl-treatment alone.

### 3.5 Eugenol modulated ionic balance in tobacco seedlings under salt stress

**Figure 7:**
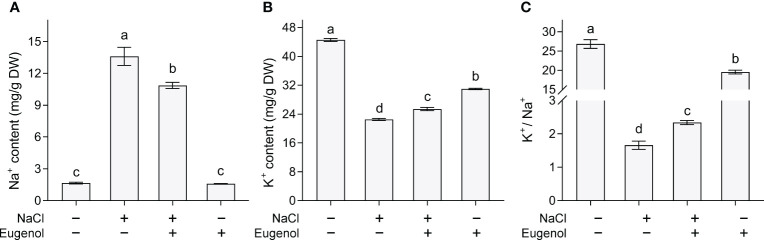
Effect of eugenol on ionic balance in tobacco seedlings under salt stress. **(A)** Na^+^ content. **(B)** K^+^ content. **(C)** The ratio of K^+^ to Na^+^. Different lowercase letters indicate significant difference among different treatments (n = 3, p < 0.05, LSD).

Na^+^ and K^+^ play negative and positive roles, respectively, in regulating plant growth under salt stress. Adding eugenol significantly decreased Na^+^ content by 20.2% and increased K^+^ content by 12.8% in NaCl-treated tobacco seedlings (**Figures 7A, B**). Salt stress decreased the ratio of K^+^/Na^+^, but adding eugenol significantly increased the ratio by 41.0% in NaCl-treated seedlings (**Figure 7C**).

**Figure 8:**
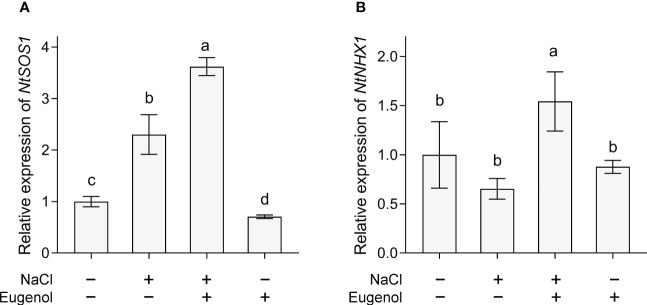
Effect of eugenol on the expression of *NtSOS1* **(A)** and *NtNHX1* **(B)** in tobacco seedlings under salt stress. Different lowercase letters indicate significant difference among different treatments (n = 3, p < 0.05, LSD).

*SOS1* and *NHX1* are two important transporters maintaining ionic homeostasis in plant cells under salt stress. Compared to NaCl treatment alone, treatment with NaCl + eugenol resulted in significant increase in the expression of *NtSOS1* and *NtNHX1* by 57.2% and 135.9%, respectively, in tobacco seedlings (**Figure 8**).

### 3.6 Hierarchical cluster analysis of eugenol-regulated physiological changes

**Figure 9:**
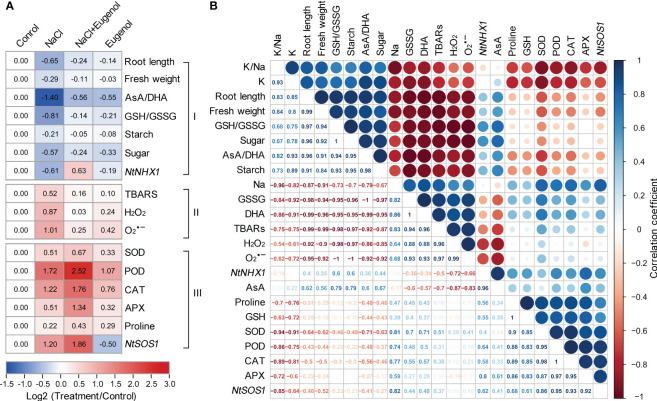
Cluster and correlation analysis among physiological parameters in tobacco seedlings under treatment of NaCl, NaCl + Eugenol, and Eugenol. **(A)** Hierarchical cluster analysis. **(B)** Pearson correlation analysis.

The hierarchical cluster analysis was performed to fully understand the changes of physiological parameters obtained above. All the parameters were clustered to three groups (I, II, and III) under the treatment of NaCl and eugenol (**Figure 9A**). The group I consisted of biomass indexes (root length and fresh weight), the content of starch and soluble sugar, AsA/DHA, GSH/GSSG, and *NtNHX1* expression. All of these parameters were beneficial for plant tolerance against salt stress. In group I, NaCl treatment resulted in the decrease in these parameters while adding eugenol enhanced them in NaCl-treated seedlings. The group II consisted of TBARS, H_2_O_2_, and O_2_^·−^, all of which were injury indexes. In group II, NaCl treatment enhanced all these three parameters while adding eugenol decreased them in NaCl-treated seedlings. Group III consisted of SOD, POD, CAT, APX, proline, and *NtSOS1* expression. All of these parameters were beneficial for plant growth under salt stress. All of these parameters were beneficial for plant tolerance against salt stress. In group III, NaCl treatment increased these parameters while adding eugenol further enhanced them in NaCl-treated seedlings (**Figure 9A**).

Pearson correlation analysis was performed to help understand the relationships among different parameters during eugenol-conferred salt tolerance (**Figure 9B**). The biomass indexes (root length and fresh weight) were positively correlated to K^+^/Na^+^, K^+^, GSH/GSSG, AsA/DHA, starch, and soluble sugar, respectively. This suggested that these parameters contributed to eugenol-promoted plant growth under salt stress. The biomass indexes were negatively correlated to Na^+^, GSSG, DHA, TBARS, H_2_O_2_, and O_2_^·−^, suggesting that these parameters contributed to salt-induced phytotoxicity. Na^+^ was positively correlated to GSSG, DHA, TBARS, H_2_O_2_, and O_2_^·−^, respectively. This suggested that Na^+^ accumulation led to oxidative injury by damaging redox balance, an effect that could be reversed by adding eugenol. *NtSOS1* was positively correlated to Na^+^, suggesting the role of *NtSOS1* in detoxifying excessive Na^+^.

## 4 Discussion

Seeking effective biostimulants with the ability of conferring plant salt tolerance is a promising approach to help crops combat salinity (Ahmad et al., 2022). Eugenol is natural compound derived from plants. Eugenol has broad antimicrobial activity (Marchese et al., 2017), but the role of eugenol in modulating plant physiology is rarely known. In this study, we revealed the novel role of eugenol in inducing salt tolerance in plants. Eugenol triggered salt tolerance by activating antioxidative capacity, adjusting osmotic balance, and modulating ionic homeostasis in tobacco seedlings.

### 4.1 Eugenol reduces ROS accumulation under salt stress

Plant cells maintain low level of ROS working as signaling molecule to regulate cell functions and plant development, but over-accumulated ROS are detrimental to plants under stress conditions (Hasanuzzaman et al., 2020). Salt stress often induces ROS accumulation, leading top membrane lipid peroxidation and cell death (Zhao et al., 2021). Eugenol promoted seedlings growth was linked to the alleviation of oxidative injury and cell death. This mainly due to the decrease in the level of cellular ROS (including both H_2_O_2_ and O_2_^·−^) in tobacco seedlings. Eugenol-restricted ROS may resulted from the enhancement of the activity of antioxidative enzymes in NaCl-treated seedlings. O_2_^·−^ is easily generated with only one electron transferred to oxygen. Therefore, O_2_^·−^ is often considered as the first ROS generated in plant cells upon stress conditions (Larosa and Remacle, 2018). SOD acts as the scavenger of O_2_^·−^ by catalyzing O_2_^·−^ to H_2_O_2_ that can be further metabolized to H_2_O by CAT, POD, and APX (Choudhury et al., 2017). Exogenous regulation of salt tolerance has been associated with the activation of antioxidant enzymes in tomato and lettuce (Patani et al., 2023; El-Salam Shalaby, 2024). In the present study, we found that salt stress induced slight increase in the activity of these enzymes. The possible reason was that salt-induced ROS accumulation act as signal to initiate enzymatic antioxidative system (Yang and Guo, 2018). However, the increase in these enzymes may not enough to scavenge large amounts of ROS induced by salt. Adding eugenol further enhanced the activity of these enzymes, which may helped further decrease ROS level in tobacco seedlings under salt stress. It has been reported that eugenol acts as antioxidant agent protecting oxidative injury by activating antioxidative enzymes in liver and lung (Yogalakshmi et al., 2010; Huang et al., 2015). It seems that eugenol can activate enzymatic antioxidative system in both plant and mammalian cells upon stress conditions.

AsA and GSH are two important components of non-enzymatic antioxidative system in plants. AsA and GSH can scavenge ROS directly through transferring electron (Briviba et al., 1997; Smirnoff, 2000). The role of GSH and AsA in inhibiting plant ROS accumulation has been identified in salt acclimation (Kumari et al., 2023). Eugenol increased the content of these two antioxidants, which helped tobacco seedlings lower endogenous ROS level under salinity stress. AsA/DHA and GSH/GSSG has been termed as redox managers for maintaining the redox balance in plant cells. Plant cells tend to maintain a favorable cellular environment at a reduced state with relatively high AsA/DHA and GSH/GSSG under normal growth conditions. Stress conditions may disrupt the redox balance to make a shift to oxidized state, which is harmful for cell function and plant development (Debolt et al., 2007; Zechmann, 2017). Eugenol significantly enhanced both AsA/DHA and GSH/GSSG in salt-treated tobacco seedlings, indicating the capability of eugenol in maintaining cell reduced state in order to combat ROS-induced oxidative stress. In addition, AsA-GSH cycle is an important pathway by coupling the transformation of AsA-DHA and GSH-GSSG. AsA-GSH pathway is one of the major pathways of antioxidative defense for detoxifying ROS in plant cells (Hasanuzzaman et al., 2019). Further studies maybe needed to possible regulation of AsA-GSH cycling by eugenol under salt stress.

It has been suggested that eugenol itself can quench ROS directly *in vitro*. And this ability has been associated with eugenol-reduced radicals in mammalian cells (Gülçin, 2011; Pérez-Rosés et al., 2016). Therefore, apart from activating antioxidative system, we speculate that the eugenol entered into plant cells may directly quench cellular ROS upon salt stress. This hypothesis should be confirmed by detecting the uptake and distribution of eugenol in plants and the *in vivo* interaction between eugenol and ROS in the future.

### 4.2 Eugenol regulates osmotic balance upon salt stress

Osmotic stress is a typical consequences of salt stress in plants. Carbohydrates (such as sugars and starch) and proline are important primary metabolites acting as osmoprotectants to combat salt-induced osmotic stress (Patel et al., 2020). Induction of these osmoregulatory metabolites has been linked to the enhancement of salt tolerance in citrus and cotton plants (Lu et al., 2023; Zhang et al., 2023a). Plant extracts could be used as potential biostimulants with the ability of inducing plant salt tolerance by priming the synthesis of soluble carbohydrates or proline (Ghezal et al., 2016; Pehlivan, 2018; Lorenzo et al., 2019; Rady et al., 2019; Desoky et al., 2020). Salt stress led to an increase in proline content and a decrease in soluble sugar and starch content in tobacco seedlings, suggesting the occurrence of osmotic stress upon salt exposure. Addition of eugenol induced the accumulation of these osmoregulatory substances in NaCl-treated seedlings, indicating the activation of osmotic adjustment. Eugenol-induced high levels of these metabolites may result from the maintenance of cell membrane integrity. Salt stress caused cell membrane damage and the leakage of these metabolite, whereas eugenol stabilized the integrity of cell membrane to prevent metabolite leakage under salt stress. Plant extracts-triggered biosynthesis of osmotic metabolites to improve salinity acclimation via modulating hormonal signaling and metabolic reprogramming (Bulgari et al., 2015; Rehman et al., 2021). In the present study, induction of osmotic metabolites accumulation is one the important strategies for eugenol-conferred salt tolerance. However, how eugenol regulates the biosynthesis and the metabolism of osmoprotectants against salt stress needs further investigation.

### 4.3 Eugenol regulates ionic balance under salt stress

Maintaining ionic balance inside of cells is important for plants’ adaption to salt stress. *SOS1*, a regulatory star of salt tolerance, works on Na^+^ detoxification by performing Na^+^ efflux out of cells (Núñez-Ramírez et al., 2012). *NHX1* has been identified as a tonoplast-located Na^+^/H^+^ antiporter, detoxifying cytosolic Na^+^ through compartmentalizing Na^+^ in vacuole (Wu et al., 2019). Thymol can inhibit Na^+^ inward flow and reduces Na^+^ content in tobacco seedlings by activating *NtSOS1* and *NtNHX1* (Xu et al., 2022). The upregulation of *NtSOS1* expression may contribute to eugenol-induced decrease in Na^+^ content in salt-treated tobacco seedlings. In addition, eugenol also up-regulated the expression of *NtNHX1*, which may facilitate the influx of Na^+^ into vacuole inside of tobacco cells. However, the distribution of Na^+^ in different organelles should be further investigated in order to confirm the possible function of eugenol in distributing intracellular Na^+^. Ca^2+^ is an essential regulator of SOS pathway that is a signaling cascade comprising SOS1, SOS2, and SOS3. The SOS3 can be activated through binding to Ca^2+^. Then Ca^2+^ activated SOS3 recruits and interacts with SOS2 to activate SOS1 (Wang et al., 2022). The Ca^2+^-SOS3-SOS2 module can also positively regulate vacuolar NHX (Zhao et al., 2021). Eugenol has been reported as an activator of Ca^2+^ channel TRPV1 (transient receptor potential vanilloid channel 1) in mammals. Eugenol can increase intercellular Ca^2+^, further activating Ca^2+^ signaling cascade in mammalian cells (Jiang et al., 2022). Therefore, it is of interest to further investigate whether eugenol regulates cytosolic Na exclusion and vacuolar Na^+^ sequestration in plants by regulating Ca^2+^ signaling.

Plant salt tolerance also involves intrinsic gaseous signaling molecules, such as H_2_S (hydrogen sulfide) and NO (nitric oxide). Both H_2_S and NO can help plants reestablish redox homeostasis and ionic balance to combat salinity (Wang et al., 2012; Lai et al., 2014). The anti-inflammatory potential of eugenol has been associated with the regulation of endogenous NO in mammals (Barboza et al., 2018). Eugenol can intensify endogenous H_2_S in rice plants against heavy metal stress (Hu et al., 2018a). Therefore, further studying the possible involvement of H_2_S and NO in eugenol-conferred salt tolerance would help reveal eugenol-primed signaling network for plant salt tolerance.

## 5 Conclusion

**Figure 10:**
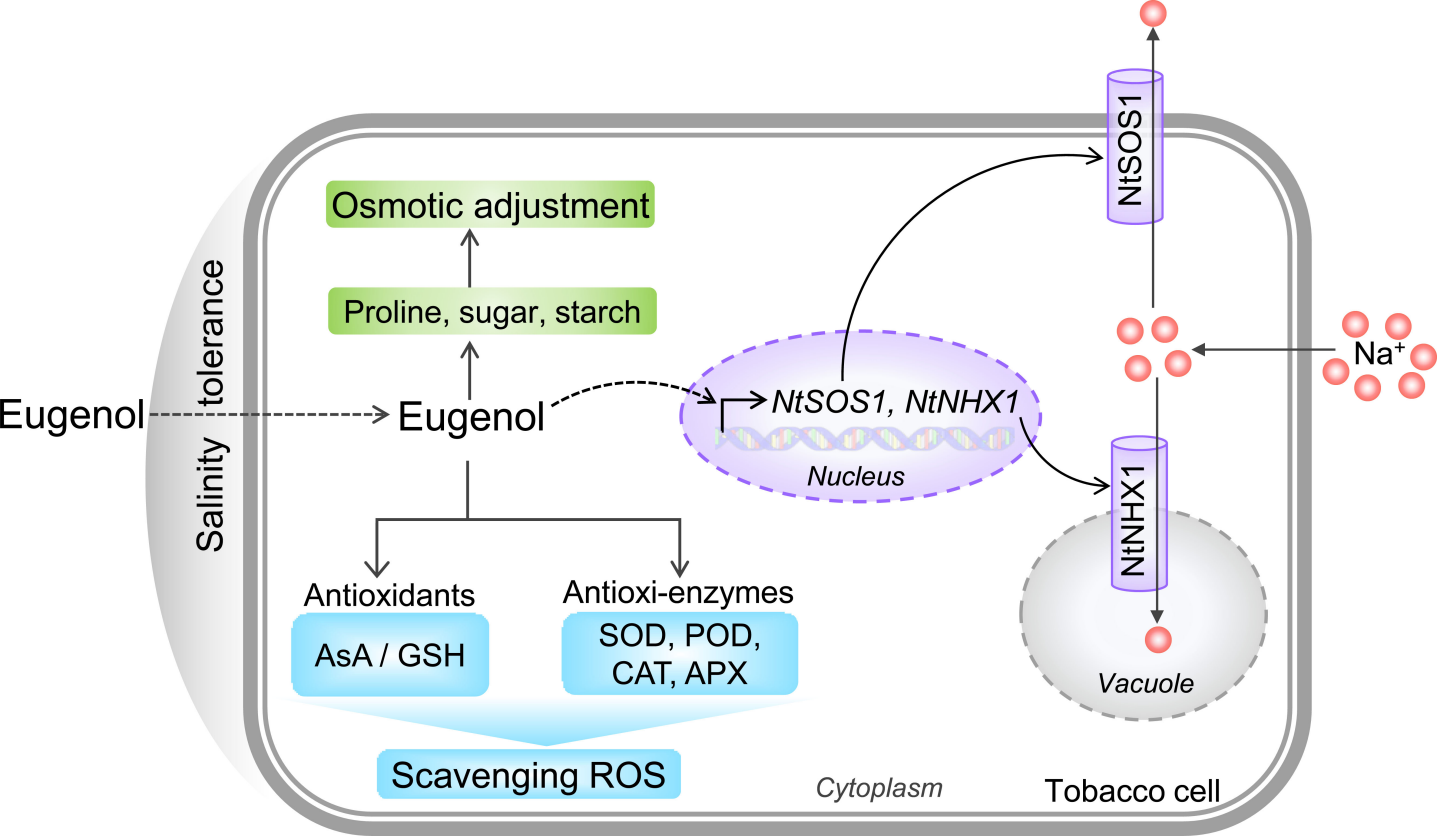
Schematic model for eugenol-facilitated three strategies that confer salinity tolerance in tobacco cells. Purple, green, and blue module represents eugenol-regulated ionic balance, osmotic adjustment, and antioxidative capacity, respectively.

In sum, the present study demonstrates that phytochemical eugenol has the ability of stimulating plant salt tolerance. Eugenol deploys three strategies to ameliorate the detrimental effect of salinity on the growth of tobacco seedlings (**Figure 10**). First, eugenol detoxifies intracellular Na^+^ by upregulating *NtSOS1* (Na^+^ efflux) and *NtNHX1* (Na^+^ sequestration in vacuole). Second, eugenol promotes osmotic adjustment by inducing the accumulation of metabolites (proline, sugar, and starch). Third, eugenol enhances antioxidative capacity by activating both antioxidants and antioxidative enzymes. The detailed molecular mechanism for eugenol-mediated reestablishment cellular redox homeostasis and ionic balance largely unknown, but our current data propose the novel function of eugenol in regulating plant salinity tolerance. These results would help develop new biostimulant for crop production in saline area, which is important for both applied and fundamental research.

## Data availability statement

The original contributions presented in the study are included in the article/supplementary material. Further inquiries can be directed to the corresponding authors.

## Author contributions

JX: Conceptualization, Investigation, Methodology, Writing – original draft. TW: Investigation, Writing – review & editing. CS: Investigation, Writing – review & editing. LP: Investigation, Writing – review & editing. JC: Data curation, Investigation, Methodology, Writing – review & editing. XH: Investigation, Writing – review & editing. TY: Investigation, Writing – review & editing. YG: Investigation, Writing – review & editing. ZL: Investigation, Writing – review & editing. LY: Conceptualization, Writing – review & editing. LZ: Conceptualization, Data curation, Investigation, Writing – review & editing.

## References

[B1] Ahmad A. Blasco B. Martos V. (2022). Combating salinity through natural plant extracts based biostimulants: A review. Front. Plant Sci. 13. doi: 10.3389/fpls.2022.862034 PMC9164010 35668803

[B2] Ali A. Petrov V. Yun D. J. Gechev T. (2023). Revisiting plant salt tolerance: novel components of the SOS pathway. Trends Plant Sci. S1360-1385 (23), 1060–1069. doi: 10.1016/j.tplants.2023.1004.1003 37117077

[B3] Al-Turki A. Murali M. Omar A. F. Rehan M. Sayyed R. Z. (2023). Recent advances in PGPR-mediated resilience toward interactive effects of drought and salt stress in plants. Front. Microbiol. 14. doi: 10.3389/fmicb.2023.1214845 PMC10565232 37829451

[B4] Aragüez I. Osorio S. Hoffmann T. Rambla J. L. Medina-Escobar N. Granell A.. (2013). Eugenol production in achenes and receptacles of strawberry fruits is catalyzed by synthases exhibiting distinct kinetics. Plant Physiol. 163 (2), 946–958. doi: 10.1104/pp.113.224352 23983228 PMC3793070

[B5] Barboza J. N. da Silva Maia Bezerra Filho C. Silva R. O. Medeiros J. V. R. de Sousa D. P. (2018). An overview on the anti-inflammatory potential and antioxidant profile of eugenol. Oxid. Med. Cell. Longev. 2018, 3957262. doi: 10.1155/2018/3957262 30425782 PMC6217746

[B6] Barragán V. Leidi E. O. Andrés Z. Rubio L. De Luca A. Fernández J. A.. (2012). Ion exchangers NHX1 and NHX2 mediate active potassium uptake into vacuoles to regulate cell turgor and stomatal function in. Arabidopsis. Plant Cell 24 (3), 1127–1142. doi: 10.1105/tpc.111.095273 22438021 PMC3336136

[B7] Briviba K. Klotz L. O. Sies H. (1997). Toxic and signaling effects of photochemically or chemically generated singlet oxygen in biological systems. Biol. Chem. 378 (11), 1259–1265. 9426185

[B8] Bulgari R. Cocetta G. Trivellini A. Vernieri P. Ferrante A. (2015). Biostimulants and crop responses: a review. Biol. Agric. Hortic. 31 (1), 1–17. doi: 10.1080/01448765.2014.964649

[B9] Chen C. Chen H. Zhang Y. Thomas H. R. Frank M. H. He Y.. (2020a). TBtools: An integrative toolkit developed for interactive analyses of big biological data. Mol. Plant 13 (8), 1194–1202. doi: 10.1016/j.molp.2020.06.009 32585190

[B10] Chen H. Lai L. Li L. Liu L. Jakada B. H. Huang Y.. (2020b). AcoMYB4, an *Ananas comosus* L. MYB transcription factor, functions in osmotic stress through negative regulation of ABA signaling. Int. J. Mol. Sci. 21 (16), 5727. doi: 10.3390/ijms21165727 32785037 PMC7460842

[B11] Choudhury F. K. Rivero R. M. Blumwald E. Mittler R. (2017). Reactive oxygen species, abiotic stress and stress combination. Plant J. 90 (5), 856–867. doi: 10.1111/tpj.13299 27801967

[B12] Debolt S. Melino V. Ford C. M. (2007). Ascorbate as a biosynthetic precursor in plants. Ann. Bot. 99 (1), 3–8. doi: 10.1093/aob/mcl236 17098753 PMC2802977

[B13] Desoky E.-S. M. El-Maghraby L. M. M. Awad A. E. Abdo A. I. Rady M. M. Semida W. M. (2020). Fennel and ammi seed extracts modulate antioxidant defence system and alleviate salinity stress in cowpea (*Vigna unguiculata*). Sci. Hortic. 272, 109576. doi: 10.1016/j.scienta.2020.109576

[B14] Drummen G. P. van Liebergen L. C. Op den Kamp J. A. Post J. A. (2002). C11-BODIPY(581/591), an oxidation-sensitive fluorescent lipid peroxidation probe: (micro)spectroscopic characterization and validation of methodology. Free Radic. Biol. Med. 33 (4), 473–490. doi: 10.1016/s0891-5849(02)00848-1 12160930

[B15] El-Salam Shalaby O. A. (2024). Moringa leaf extract increases tolerance to salt stress, promotes growth, increases yield, and reduces nitrate concentration in lettuce plants. Sci. Hortic-Amsterdam 325, 112654. doi: 10.1016/j.scienta.2023.112654

[B16] Gao Z. Zhang J. Zhang J. Zhang W. Zheng L. Borjigin T.. (2022). Nitric oxide alleviates salt-induced stress damage by regulating the ascorbate-glutathione cycle and Na^+^/K^+^ homeostasis in *Nitraria tangutorum* Bobr. Plant Physiol. Biochem. 173, 46–58. doi: 10.1016/j.plaphy.2022.01.017 35093694

[B17] Ge L. Yang X. Liu Y. Tang H. Wang Q. Chu S.. (2023). Improvement of seed germination under salt stress via overexpressing *Caffeic Acid O-methyltransferase 1 (SlCOMT1)* in *Solanum lycopersicum* L. Int. J. Mol. Sci. 24 (1), 734. doi: 10.3390/ijms24010734 36614180 PMC9821337

[B18] Ghezal N. Rinez I. Sbai H. Saad I. Farooq M. Rinez A.. (2016). Improvement of *Pisum sativum* salt stress tolerance by bio-priming their seeds using *Typha angustifolia* leaves aqueous extract. S. Afr. J. Bot. 105, 240–250. doi: 10.1016/j.sajb.2016.04.006

[B19] Gill S. S. Tuteja N. (2010). Reactive oxygen species and antioxidant machinery in abiotic stress tolerance in crop plants. Plant Physiol. Biochem. 48 (12), 909–930. doi: 10.1016/j.plaphy.2010.08.016 20870416

[B20] Guijarro-Real C. Adalid-Martinez A. M. Gregori-Montaner A. Prohens J. Rodriguez-Burruezo A. Fita A. (2020). Factors affecting germination of *Diplotaxis erucoides* and their effect on selected quality properties of the germinated products. Sci. Hortic-Amsterdam 261, 109013. doi: 10.1016/j.scienta.2019.109013

[B21] Gülçin İ. (2011). Antioxidant activity of eugenol: A structure–activity relationship study. J. Med. Food 14 (9), 975–985. doi: 10.1089/jmf.2010.0197 21554120

[B22] Hasanuzzaman M. Bhuyan M. H. M. B. Anee T. I. Parvin K. Nahar K. Mahmud J. A.. (2019). Regulation of ascorbate-glutathione pathway in mitigating oxidative damage in plants under abiotic stress. Antioxidants 8 (9), 384. doi: 10.3390/antiox8090384 31505852 PMC6770940

[B23] Hasanuzzaman M. Bhuyan M. Parvin K. Bhuiyan T. F. Anee T. I. Nahar K.. (2020). Regulation of ROS metabolism in plants under environmental stress: A review of recent experimental evidence. Int. J. Mol. Sci. 21 (22), 8695. doi: 10.3390/ijms21228695 33218014 PMC7698618

[B24] Hasanuzzaman M. Raihan M. R. H. Masud A. A. C. Rahman K. Nowroz F. Rahman M.. (2021). Regulation of reactive oxygen species and antioxidant defense in plants under salinity. Int. J. Mol. Sci. 22 (17), 9326. doi: 10.3390/ijms22179326 34502233 PMC8430727

[B25] Hu L. Li H. Huang S. Wang C. Sun W.-J. Mo H.-Z.. (2018a). Eugenol confers cadmium tolerance via intensifying endogenous hydrogen sulfide signaling in *Brassica rapa* . J. Agric. Food Chem. 66 (38), 9914–9922. doi: 10.1021/acs.jafc.8b03098 30188702

[B26] Hu Q. Zhou M. Wei S. (2018b). Progress on the antimicrobial activity research of clove oil and eugenol in the food antisepsis field. J. Food Sci. 83 (6), 1476–1483. doi: 10.1111/1750-3841.14180 29802735

[B27] Huang X. Liu Y. Lu Y. Ma C. (2015). Anti-inflammatory effects of eugenol on lipopolysaccharide-induced inflammatory reaction in acute lung injury via regulating inflammation and redox status. Int. Immunopharmacol. 26 (1), 265–271. doi: 10.1016/j.intimp.2015.03.026 25863235

[B28] Jia C. Cao D. Ji S. Zhang X. Muhoza B. (2020). Tannic acid-assisted cross-linked nanoparticles as a delivery system of eugenol: The characterization, thermal degradation and antioxidant properties. Food Hydrocolloid. 104, 105717. doi: 10.1016/j.foodhyd.2020.105717

[B29] Jiang Y. Feng C. Shi Y. Kou X. Le G. (2022). Eugenol improves high-fat diet/streptomycin-induced type 2 diabetes mellitus (T2DM) mice muscle dysfunction by alleviating inflammation and increasing muscle glucose uptake. Biochem. Biophys. Res. Commun. 9. doi: 10.3389/fnut.2022.1039753 PMC9681568 36424928

[B30] Kellermeier F. Chardon F. Amtmann A. (2013). Natural variation of arabidopsis root architecture reveals complementing adaptive strategies to potassium starvation. Plant Physiol. 161 (3), 1421–1432. doi: 10.1104/pp.112.211144 23329148 PMC3585606

[B31] Koeduka T. Fridman E. Gang D. R. Vassão D. G. Jackson B. L. Kish C. M.. (2006). Eugenol and isoeugenol, characteristic aromatic constituents of spices, are biosynthesized via reduction of a coniferyl alcohol ester. Proc. Natl. Acad. Sci. U.S.A. 103 (26), 10128–10133. doi: 10.1073/pnas.0603732103 16782809 PMC1502517

[B32] Kumari S. Kaur H. Jain A. Hussain S. J. Siddiqui M. H. Khan M. I. R. (2023). Hydrogen sulfide modulates ascorbate-glutathione system, osmolytes production, nutrient content and yield responses under salt stress in wheat. S. Afr. J. Bot. 160, 295–308. doi: 10.1016/j.sajb.2023.07.022

[B33] Lai D. Mao Y. Zhou H. Li F. Wu M. Zhang J.. (2014). Endogenous hydrogen sulfide enhances salt tolerance by coupling the reestablishment of redox homeostasis and preventing salt-induced K^+^ loss in seedlings of *Medicago sativa* . Plant Sci. 225, 117–129. doi: 10.1016/j.plantsci.2014.06.006 25017167

[B34] Larosa V. Remacle C. (2018). Insights into the respiratory chain and oxidative stress. Biosci. Rep. 38 (5), BSR20171492. doi: 10.1042/bsr20171492 30201689 PMC6167499

[B35] Liu M. H. Lv Y. Cao B. L. Chen Z. J. Xu K. (2023). Physiological and molecular mechanism of ginger *Zingiber officinale* Roscoe) seedling response to salt stress. Front. Plant Sci. 14. doi: 10.3389/fpls.2023.1073434 PMC10064006 37008470

[B36] Lorenzo P. Souza-Alonso P. Guisande-Collazo A. Freitas H. (2019). Influence of *Acacia dealbata* Link bark extracts on the growth of *Allium cepa* L. plants under high salinity conditions. J. Sci. Food Agric. 99 (8), 4072–4081. doi: 10.1002/jsfa.9637 30761550

[B37] Lu K. Yan L. Riaz M. Babar S. Hou J. Zhang Y.. (2023). “Exogenous boron alleviates salt stress in cotton by maintaining cell wall structure and ion homeostasis”. Plant Physiol. Bioch 201, 107858. doi: 10.1016/j.plaphy.2023.107858 37390694

[B38] Luo S. L. Liu Z. C. Wan Z. L. He X. X. Lv J. Yu J. H.. (2023). Foliar spraying of NaHS alleviates cucumber salt stress by maintaining Na^+^/K^+^ balance and activating salt tolerance signaling pathways. Plants-Basel 12 (13), 2450. doi: 10.3390/plants12132450 37447010 PMC10346887

[B39] Ma L. Liu X. H. Lv W. J. Yang Y. Q. (2022). Molecular mechanisms of plant responses to salt stress. Front. Plant Sci. 13. doi: 10.3389/fpls.2022.934877 PMC9271918 35832230

[B40] Mansour M. M. F. Ali E. F. (2017). Evaluation of proline functions in saline conditions. Phytochemistry 140, 52–68. doi: 10.1016/j.phytochem.2017.04.016 28458142

[B41] Marchese A. Barbieri R. Coppo E. Orhan I. E. Daglia M. Nabavi S. F.. (2017). Antimicrobial activity of eugenol and essential oils containing eugenol: A mechanistic viewpoint. Crit. Rev. Microbiol. 43 (6), 668–689. doi: 10.1080/1040841X.2017.1295225 28346030

[B42] Morcia C. Malnati M. Terzi V. (2011). *In vitro* antifungal activity of terpinen-4-ol, eugenol, carvone, 1,8-cineole (eucalyptol) and thymol against mycotoxigenic plant pathogens. Food Addit. Contam. Part A 29 (3), 415–422. doi: 10.1080/19440049.2011.643458 22257275

[B43] Negrão S. Schmöckel S. M. Tester M. (2017). Evaluating physiological responses of plants to salinity stress. Ann. Bot. 119 (1), 1–11. doi: 10.1093/aob/mcw191 27707746 PMC5218372

[B44] Núñez-Ramírez R. Sánchez-Barrena M. J. Villalta I. Vega J. F. Pardo J. M. Quintero F. J.. (2012). Structural insights on the plant Salt-Overly-Sensitive 1 (SOS1) Na^+^/H^+^ antiporter. J. Mol. Biol. 424 (5), 283–294. doi: 10.1016/j.jmb.2012.09.015 23022605

[B45] Olea A. F. Bravo A. Martínez R. Thomas M. Sedan C. Espinoza L.. (2019). Antifungal activity of eugenol derivatives against *Botrytis cinerea* . Molecules 24 (7), 1239. doi: 10.3390/molecules24071239 30934962 PMC6479685

[B46] Patani A. Prajapati D. Ali D. Kalasariya H. Yadav V. K. Tank J.. (2023). Evaluation of the growth-inducing efficacy of various *Bacillus* species on the salt-stressed tomato (Lycopersicon esculentum Mill.). Front. Plant Sci. 14. doi: 10.3389/fpls.2023.1168155 PMC10089305 37056512

[B47] Patel M. K. Kumar M. Li W. Luo Y. Burritt D. J. Alkan N.. (2020). Enhancing salt tolerance of plants: From metabolic reprogramming to exogenous chemical treatments and molecular approaches. Cells 9 (11), 2492. doi: 10.3390/cells9112492 33212751 PMC7697626

[B48] Pehlivan N. (2018). Salt stress relief potency of whortleberry extract biopriming in maize. 3 Biotech. 8 (2), 89. doi: 10.1007/s13205-018-1113-6 PMC5796940 29430351

[B49] Pérez-Rosés R. Risco E. Vila R. Peñalver P. Cañigueral S. (2016). Biological and nonbiological antioxidant activity of some essential oils. J. Agric. Food Chem. 64 (23), 4716–4724. doi: 10.1021/acs.jafc.6b00986 27214068

[B50] Quamruzzaman M. Manik S. M. N. Shabala S. Zhou M. (2021). Improving performance of salt-grown crops by exogenous application of plant growth regulators. Biomolecules 11 (6), 788. doi: 10.3390/biom11060788 34073871 PMC8225067

[B51] Rady M. M. Desoky E. S. M. Elrys A. S. Boghdady M. S. (2019). Can licorice root extract be used as an effective natural biostimulant for salt-stressed common bean plants? S. Afr. J. Bot. 121, 294–305. doi: 10.1016/j.sajb.2018.11.019

[B52] Rehman H.U. Alharby H. F. Bamagoos A. A. Abdelhamid M. T. Rady M. M. (2021). Sequenced application of glutathione as an antioxidant with an organic biostimulant improves physiological and metabolic adaptation to salinity in wheat. Plant Physiol. Biochem. 158, 43–52. doi: 10.1016/j.plaphy.2020.11.041 33296845

[B53] Shrivastava P. Kumar R. (2015). Soil salinity: A serious environmental issue and plant growth promoting bacteria as one of the tools for its alleviation. Saudi J. Biol. Sci. 22 (2), 123–131. doi: 10.1016/j.sjbs.2014.12.001 25737642 PMC4336437

[B54] Smirnoff N. (2000). Ascorbic acid: metabolism and functions of a multi-facetted molecule. Curr. Opin. Plant Biol. 3 (3), 229–235. doi: 10.1016/S1369-5266(00)80070-9 10837263

[B55] Song R. F. Li T. T. Liu W. C. (2021). Jasmonic acid impairs arabidopsis seedling salt stress tolerance through MYC2-Mediated repression of *CAT2* expression. Front. Plant Sci. 12. doi: 10.3389/fpls.2021.730228 PMC8569249 34745163

[B56] Sun G. Geng S. Zhang H. Jia M. Wang Z. Deng Z.. (2022). Matrilineal empowers wheat pollen with haploid induction potency by triggering postmitosis reactive oxygen species activity. New Phytol. 233 (6), 2405–2414. doi: 10.1111/nph.17963 35015909

[B57] Sun H. Sun X. Wang H. Ma X. (2020). Advances in salt tolerance molecular mechanism in tobacco plants. Hereditas 157, 5. doi: 10.1186/s41065-020-00118-0 32093781 PMC7041081

[B58] Taleuzzaman M. Jain P. Verma R. Iqbal Z. Mirza A. M. (2021). Eugenol as a potential drug candidate: A review. Curr. Top. Med. Chem. 21 (20), 1804–1815. doi: 10.2174/1568026621666210701141433 34218781

[B59] van Zelm E. Zhang Y. Testerink C. (2020). Salt tolerance mechanisms of plants. Annu. Rev. Plant Biol. 71 (1), 403–433. doi: 10.1146/annurev-arplant-050718-100005 32167791

[B60] Wang C.-F. Han G.-L. Yang Z.-R. Li Y.-X. Wang B.-S. (2022). Plant salinity sensors: Current understanding and future directions. Front. Plant Sci. 13. doi: 10.3389/fpls.2022.859224 PMC9022007 35463402

[B61] Wang Y. Li L. Cui W. Xu S. Shen W. Wang R. (2012). Hydrogen sulfide enhances alfalfa (*Medicago sativa*) tolerance against salinity during seed germination by nitric oxide pathway. Plant Soil 351 (1), 107–119. doi: 10.1007/s11104-011-0936-2

[B62] Wang B. K. Wang J. Yang T. Wang J. X. Dai Q. Zhang F. L.. (2023a). The transcriptional regulatory network of hormones and genes under salt stress in tomato plants (*Solanum lycopersicum* L.). Front. Plant Sci. 14. doi: 10.3389/fpls.2023.1115593 PMC9939653 36814758

[B63] Wang Z. Zhang W. Huang W. Biao A. Lin S. Wang Y.. (2023b). Salt stress affects the fruit quality of *Lycium ruthenicum* Murr. Ind. Crop Prod. 193, 116240. doi: 10.1016/j.indcrop.2023.116240

[B64] Wang B. Zhang J. Pei D. Yu L. (2021). Combined effects of water stress and salinity on growth, physiological, and biochemical traits in two walnut genotypes. Physiol. Plant 172 (1), 176–187. doi: 10.1111/ppl.13316 33314146

[B65] Wei T. Simko V. (2017). "R package "corrplot": Visualization of a correlation matrix (Version 0.84)". Available at: https://github.com/taiyun/corrplot.

[B66] Wu H. Shabala L. Zhou M. Su N. Wu Q. Ul-Haq T.. (2019). Root vacuolar Na^+^ sequestration but not exclusion from uptake correlates with barley salt tolerance. Plant J. 100 (1), 55–67. doi: 10.1111/tpj.14424 31148333

[B67] Xiao F. Zhou H. P. (2023). Plant salt response: Perception, signaling, and tolerance. Front. Plant Sci. 13. doi: 10.3389/fpls.2022.1053699 PMC9854262 36684765

[B68] Xu L. Song J. Q. Wang Y. L. Liu X. H. Li X. L. Zhang B.. (2022). Thymol improves salinity tolerance of tobacco by increasing the sodium ion efflux and enhancing the content of nitric oxide and glutathione. BMC Plant Biol. 22 (1), 31. doi: 10.1186/s12870-021-03395-7 35027009 PMC8756686

[B69] Yang Y. Guo Y. (2018). Unraveling salt stress signaling in plants. J. Integr. Plant Biol. 60 (9), 796–804. doi: 10.1111/jipb.12689 29905393

[B70] Ye X. Ling T. Xue Y. Xu C. Zhou W. Hu L.. (2016). Thymol mitigates cadmium stress by regulating glutathione levels and reactive oxygen species homeostasis in tobacco seedlings. Molecules 21 (10), 1339. doi: 10.3390/molecules21101339 27754435 PMC6273743

[B71] Ye X.-F. Xue Y. Ling T. Wang Y. Yu X.-N. Cheng C.. (2017). Cinnamaldehyde ameliorates cadmium-inhibited root elongation in tobacco seedlings via decreasing endogenous hydrogen sulfide production. Molecules 22 (1), 15. doi: 10.3390/molecules22010015 PMC6155710 28029133

[B72] Yogalakshmi B. Viswanathan P. Anuradha C. V. (2010). Investigation of antioxidant, anti-inflammatory and DNA-protective properties of eugenol in thioacetamide-induced liver injury in rats. Toxicology 268 (3), 204–212. doi: 10.1016/j.tox.2009.12.018 20036707

[B73] Yu Z. Duan X. Luo L. Dai S. Ding Z. Xia G. (2020). How plant hormones mediate salt stress responses. Trends. Plant Sci. 25 (11), 1117–1130. doi: 10.1016/j.tplants.2020.06.008 32675014

[B74] Zechmann B. (2017). Diurnal changes of subcellular glutathione content in *Arabidopsis thaliana* . Biol. Plant 61 (4), 791–796. doi: 10.1007/s10535-017-0729-4

[B75] Zhang W. B. He X. L. Chen X. J. Han H. W. Shen B. R. Diao M.. (2023b). Exogenous selenium promotes the growth of salt-stressed tomato seedlings by regulating ionic homeostasis, activation energy allocation and CO_2_ assimilation. Front. Plant Sci. 14. doi: 10.3389/fpls.2023.1206246 PMC10352764 37469781

[B76] Zhang M. M. Li X. Y. Wang X. L. Feng J. P. Zhu S. P. (2023a). Potassium fulvic acid alleviates salt stress of citrus by regulating rhizosphere microbial community, osmotic substances and enzyme activities. Front. Plant Sci. 14. doi: 10.3389/fpls.2023.1161469 PMC10076529 37035078

[B77] Zhang W. D. Wang P. Bao Z. Ma Q. Duan L. J. Bao A. K.. (2017). SOS1, HKT1;5, and NHX1 synergistically modulate Na^+^ homeostasis in the halophytic grass *Puccinellia tenuiflora* . Front. Plant Sci. 8. doi: 10.3389/fpls.2017.00576 PMC5390037 28450879

[B78] Zhao M. Y. Jin J. Y. Wang J. M. Gao T. Luo Y. Jing T. T.. (2022). Eugenol functions as a signal mediating cold and drought tolerance via UGT71A59-mediated glucosylation in tea plants. Plant J. 109 (6), 1489–1506. doi: 10.1111/tpj.15647 34931743

[B79] Zhao S. Zhang Q. Liu M. Zhou H. Ma C. Wang P. (2021). Regulation of plant responses to salt stress. Int. J. Mol. Sci. 22 (9), 4609. doi: 10.3390/ijms22094609 33924753 PMC8125386

